# Computer-Based Cognitive Training in Aging

**DOI:** 10.3389/fnagi.2016.00313

**Published:** 2016-12-20

**Authors:** Blanka Klimova

**Affiliations:** Department of Applied Linguistics, Faculty of Informatics and Management, University of Hradec KraloveHradec Kralove, Czechia

**Keywords:** cognitive decline, intervention, memory, older people, online training, randomized controlled clinical trials

## Abstract

At present there is a rapid growth of aging population groups worldwide, which brings about serious economic and social problems. Thus, there is considerable effort to prolong the active life of these older people and keep them independent. The purpose of this mini review is to explore available clinical studies implementing computer-based cognitive training programs as intervention tools in the prevention and delay of cognitive decline in aging, with a special focus on their effectiveness. This was done by conducting a literature search in the databases Web of Science, Scopus, MEDLINE and Springer, and consequently by evaluating the findings of the relevant studies. The findings show that computerized cognitive training can lead to the improvement of cognitive functions such as working memory and reasoning skills in particular. However, this training should be performed over a longer time span since a short-term cognitive training mainly has an impact on short-term memory with temporary effects. In addition, the training must be intense to become effective. Furthermore, the results indicate that it is important to pay close attention to the methodological standards in future clinical studies.

## Introduction

At present people’s life expectancy is increasing. Therefore there is a substantial rise in the number of aging population groups, which causes significant social and economic problems. Thus, there is considerable effort to keep these older people active as long as possible. One of the main features of aging is worsening of cognitive functions, especially working memory, which is considered to be a healthy part of aging, but together with other neuropsychological deficits, it can also mark the first stages of a dementing neurodegenerative disease, most commonly Alzheimer’s disease (Klimova et al., 2015a). Dementia is one of the main causes of incapability and dependency of older people. As Kirshner (2014) states, dementia is a syndrome of deterioration of cognitive functions that interfere with everyday life. This damage impedes communication between brain cells and this consequently results in worsening of cognitive, behavioral, motor control, and other functions (Klimova and Kuca, 2016). The most common symptoms of dementia include loss of memory, orientation problems, impaired communication skills, depression, behavioral changes and confusion.

However, it has been argued that through regular cognitive training, older people can maintain or even enhance their cognitive functions (Borella et al., 2013; Karbach and Schubert, 2013). This would be based on increased functional abilities and cognitive fitness, partly compensating for the pathological incurring in the aging brain. Although meta-analytic reviews differ in their views on the efficacy of cognitive training programs (cf. Gross et al., 2012; Kueider et al., 2012; Melby-Lervåg and Hulme, 2013, 2016; Redick et al., 2013; Karbach and Verhaeghen, 2014; Melby-Lervåg et al., 2016), the findings of the clinical trials (Borella et al., 2013, 2014; Rebok and Ball, 2014; Zinke et al., 2014; Corbett et al., 2015; Rizkalla, 2015) indicate that cognitive training, especially memory training might be a good intervention tool in the maintenance or even in the improvement of cognitive competences of older people. For example, the study by Zinke et al. (2014) observed that cognitive plasticity was preserved even in the old age and that also a short-term cognitive training may lead to partly specific training and transfer effects. Borella et al. (2013) note that there is still room for older people to improve their working memory skills since the findings of their study show that working memory training programs generate persistent benefits, particularly in the verbal working memory tasks.

In fact, cognitive training has gained considerable popularity in the past two decades (Walton et al., 2015). It has been argued to improve working memory capacity and cognitive skills and functions of people with working memory deficits (Morrison and Chein, 2011; Rebok and Ball, 2014). Cognitive training can be administered in different ways; it can be process-based, which includes repetitive, drill-like training on specific tasks, or more strategic, individualized intervention, based on memory formation strategies such as the method of loci or mnemonic story (Gross et al., 2012; Walton et al., 2015).

Most recently, with the penetration of technologies in all spheres of human activities, technological devices have started to play a significant role in cognitive training since such training can be done at any time and accessed from anywhere. In addition, it can be personalized to people’s own needs (Klimova et al., 2015b, 2016; Maresova and Klimova, 2015). This approach is also more cost-effective since people can do it at home. And such training programs can be more easily disseminated among a wide range of people (Klimova and Maresova, 2016). Furthermore, research studies (Hernandez-Encuentra et al., 2009; Sayago et al., 2011) have proved that older people in their 60s and 70s are nowadays much more digitally aware than they were 10 years ago. Kueider et al. (2012) also note that older people do not have to be necessarily technologically savvy to benefit from computer-based training programs.

Altogether there are three general approaches to enhance cognitive functions with the help of a computer. These include brain training programs, working memory training programs, and video game training programs (Boot and Kramer, 2014). The brain training programs usually focus on the improvement of the speed and accuracy of perceptual processes, aiming at improved attention, episodic memory, executive function, reasoning, speech and language, or visual-spatial skills. At present there are five well tested brain training applications, which are as follows: Elevate – a cognitive training tool to build communication and analytical skills (Elevate, 2014); Lumosity – a series of online games that is targeted at the improvement of memory, speed, problem solving, attention, flexibility, which may help with remembering names and driving better (Lumosity, 2016); Fit Brains – an application which focuses on the enhancement of mental performance through games and has a similar effect as Lumosity (Fit Brains, 2016); Brain HQ developed by Posit Science company, providing a series of training exercises, which can improve the ability to process visual scenes, working memory or cognitive flexibility (Brain Training, 2016); or Brain workshop – an application which aims at the improvement of the short-term memory and fluid intelligence (Brain Workshop, 2016). The working memory programs are aimed at the enhancement of working memory, which is a fundamental intellectual faculty. It represents a system that keeps multiple pieces of transitory information in the mind, information that is needed for different ongoing tasks. In addition, a study by Anguera et al. (2013) indicates that video game training programs can be a powerful tool in the improvement of cognitive functions such as interference resolution, working memory or sustained memory. Nowadays, there is a boom of cognitive exercise products which can be accessed online (Fernandez, 2011), but there is still a lack of the proof of their efficacy (Kueider et al., 2012). This is also confirmed by Melby-Lervåg and Hulme (2013) who claim that the well-known commercial, computer-based training programs such as CogMed, Jungle Memory, or Cognifit are not based on any thorough task analysis or theoretical explanation of the training mechanism responsible for the improvement of working memory capacity.

The purpose of this mini review is to explore available clinical studies implementing computer-based cognitive training programs as intervention tools in the prevention and delay of cognitive decline in aging, with a special focus on their effectiveness.

## Methods

The methodology of this mini review study is based on Kurz and van Baelen (2004) and Moher et al. (2009). Thus, the relevant literature was searched and the findings of different studies exploring computer-based cognitive training, especially memory training, were examined. Research studies were selected on the basis of the research topics (i.e., computer-based cognitive training AND older people, computer-based memory training AND older people, online cognitive training AND older people, online cognitive training AND older people) found in research studies in peer-review English written articles from the databases Web of Science, Springer, Scopus, and MEDLINE from the period of 2013 up to the present time. The research studies were then classified according to their relevancy. Altogether, 382 studies were found via the database search and 57 studies via other sources, which included conference proceedings and books outside the scope of the databases described above. After a thorough review of the titles and abstracts and their duplication of the selected studies, only 37 studies remained for the full-text analysis. After that, only six randomized clinical studies were identified. A study was included if it matched the corresponding period, i.e., from 2013 up to 2016; the period is limited to these years only since till 2013 several review studies on cognitive training, including the use technologies, had been already published (e.g., Gross et al., 2012; or Kueider et al., 2012). Furthermore, the study was included if it only involved older people aged 50+, either fully healthy individuals or just with mild cognitive impairment, aimed at cognitive, especially memory training, and were written in English. Theoretical articles, review articles and book chapters were excluded, as well as the research studies examining neuropsychological software programs. Nevertheless, the review articles and other descriptive research studies were then used in other parts of this manuscript (i.e., Introduction or Discussion) in order to describe and compare the findings.

**FIGURE 1:**
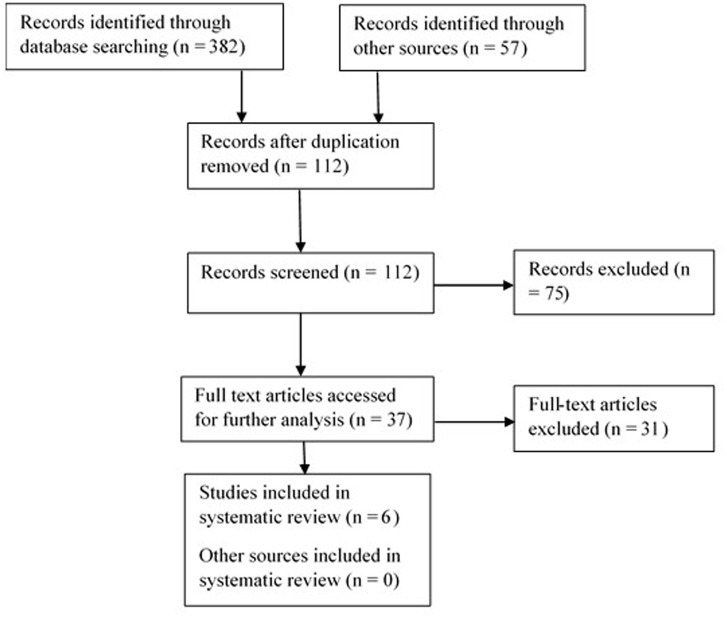
**Flowchart of the review procedure**.

**Figure 1** below then illustrates the selection procedure, which was done in the following four steps:

(i) Identification (identification of the key words and consequently, available relevant sources);
(ii) Duplication check;
(iii) Assessment of relevancy (verification on the basis of abstracts whether the selected study corresponds to the set goal; after the exclusion of such studies, 37 sources were analyzed and 31 eventually excluded).

## Findings

Altogether six randomized clinical studies were eventually identified in this mini review. Five clinical studies were randomized controlled clinical trials (McAvinue et al., 2013; Corbett et al., 2015; Rose et al., 2015; Walton et al., 2015; Hyer et al., 2016), and one study included two randomly allotted intervention groups (Bozoki et al., 2013). All of them applied only computerized cognitive training. Therefore, other randomized controlled clinical trials in the field such as Borella et al. (2013, 2014), Rebok and Ball (2014), Zinke et al. (2014) and Rizkalla (2015) were excluded. They are summarized in alphabetical order of their first author in **Table 1** below.

**Table 1:** Overview of the randomized clinical studies on computer-based cognitive training in the elderly.

Study	Type of intervention	No. of subjects	Trial period	Findings	Limitations
Bozoki et al., 2013	Computer-based cognitive exercise program (online games), active group	60 older subjects (age 60–80)	6 weeks	No effects, only improvements on games.	A small sample size; a short-term period of the trial; no control group; low program intensity.
Corbett et al., 2015	Cognitive training program, active control group	2,192 older subjects; mean age 65	6 months	Improved cognition, particularly the reasoning skills, which was evident already from week six.	Only people with computer access were included into the trial; people with higher levels of education; retention strategies need to be improved.
Hyer et al., 2016	Cognitive training program CogMed for the intervention group and Sham for the active control group	68 older subjects with Mild Cognitive Impairment (MCI)	7 weeks	Working memory of both groups was enhanced, but the CogMed group had higher ratings of satisfaction.	A small sample size; a short-term period of the trial; a lack of the program intensity.
McAvinue et al., 2013	Computerized program, passive control group	36 healthy older subjects (age 64–79)	A 5-week training period + a 6-month follow up	The results confirmed enhanced short-term memory, together with transfer of training gains to a long-term episode memory tasks.	A lack of inclusion of a measure of visuospatial short-term or working memory; non-adaptive version of the training program for the control group; a small sample size.
Rose et al., 2015	Virtual Week training program, active control group	59 healthy older subjects (mean age 67.4)	1 month (12 sessions, each 1 h long)	Improved prospective memory; transfer to real-world settings, which was reflected in participants’ daily activities.	A small sample size; a short-term period of the trial; a lack of effective strategies used by participants.
Walton et al., 2015	Cognitive training program, active control group	28 healthy older subjects (mean age 64.18)	28 days	Improved performance in multiple measures of processing speed; visual working memory can be enhanced over a short period of computerized cognitive training.	A lack of the follow up assessment; a small sample size; a short-term period.

## Discussion Of The Findings

The findings of the studies in **Table 1** indicate that computerized cognitive training can lead to the improvement of cognitive functions such as reasoning skills (Corbett et al., 2015), short-term memory (McAvinue et al., 2013), working memory (Hyer et al., 2016), processing speed and visual working memory (Walton et al., 2015) in particular. However, this training should be performed over a longer time span since a short-term cognitive training mainly has an impact on short-term memory with temporary effects (McAvinue et al., 2013; Walton et al., 2015). In addition, the training must be intense to become effective (cf. Zelinski et al., 2011; Haesner et al., 2015). The review study on computerized cognitive training conducted by Lampit et al. (2014) shows that computer-based cognitive training should be performed for more than 30 min since synaptic plasticity is possible after 30–60 min of stimulation (Luscher et al., 2000). Nevertheless, they also point out to the fact that this training should be done only three times a week, otherwise it has a reverse effect. In comparison with the findings described above, Lampit et al. (2014) claim that computer-based cognitive training has only moderate effects in improving cognitive functioning in healthy older individuals. In addition, its efficacy varies across cognitive domains and is determined by design choices. This also supports the claim of Melby-Lervåg and Hulme (2013, 2016) and Melby-Lervåg et al. (2016) that there are important differences in methodologies used in the randomized controlled clinical trials. Methodological issues such as the use of passive control groups or the failure to consider baseline differences between the groups may lead to overestimation of the training effects, seriously threatening the validity of the findings.

Although the critical arguments present above raise doubts concerning the efficacy of computer-based cognitive training, there is ongoing work to develop computer-based cognitive programs for older people since clinical studies indicate that these training may generate transfer effects, specifically near-transfer effects, both in healthy older individuals and older people with MCI (Stepankova et al., 2012; Flak et al., 2014). However, the results of this mini review also indicate that there is still a lack of larger sample longitudinal randomized controlled clinical trials in computer-based cognitive training among healthy aging population groups.

There are also other issues that are worth considering when developing computerized cognitive training programs for the elderly. For example, if older people have a negative attitude to the use of computer programs, they can use the so-called stress-free devices such as TV instead. The study by Shatil et al. (2014) shows how older people’s working memory improved when they were exposed to the cognitive training provided through an interactive TV. In fact, studies (cf. Wolfson and Kraiger, 2014) indicate that there is a need for age-specific computer-based instructional design and formats.

Apart from cognitive training, older people should conduct other activities in order to delay cognitive decline. Klimova and Kuca (2015) present three main activities this population group should do in order to prevent or delay aging processes. These involve physical activities, cognitive training and adherence to the Mediterranean diet. However, as it has been already stated above, all these non-invasive approaches must be performed intensively and frequently in order to efficiently delay the cognitive decline or improve cognitive competences. The rationale for this is that intense physical activities can raise vascular endothelial growth factor in the brain of younger people (Li et al., 2011). Radak et al. (2013) also claim that physical activities can improve the resistance against oxidative stress, help to restore the brain and maintain cognitive function.

## Conclusion

Based on the findings of this mini review, computer-based cognitive training predominantly targeted at healthy elderly can be beneficial in several ways: it is a non-invasive treatment, it can be tailored-made to older people’s needs, it is cost-effective and can be made widely available, and it seems to be an effective intervention tool, especially as far as the short-term specific trainings with near-transfer effects are concerned. Nevertheless, it is important to pay close attention to the methodological standards in future clinical studies. In addition, more randomized controlled clinical trials should be conducted to establish efficacy of these computer-based training programs in the prevention and delay of cognitive decline among healthy older individuals.

## Author Contributions

BK has prepared and written this manuscript on her own.

## Conflict of Interest Statement

The author declares that the research was conducted in the absence of any commercial or financial relationships that could be construed as a potential conflict of interest.
